# Multimodal synchrotron X-ray fluorescence imaging reveals elemental distribution in seeds and seedlings of the Zn–Cd–Ni hyperaccumulator *Noccaea caerulescens*

**DOI:** 10.1093/mtomcs/mfac026

**Published:** 2022-06-23

**Authors:** Antony van der Ent, Martin D de Jonge, Guillaume Echevarria, Mark G M Aarts, Jolanta Mesjasz-Przybyłowicz, Wojciech J Przybyłowicz, Dennis Brueckner, Hugh H Harris

**Affiliations:** Centre for Mined Land Rehabilitation, Sustainable Minerals Institute, The University of Queensland, St Lucia 4072, Australia; Australian Synchrotron, ANSTO, Clayton 3168, Australia; Laboratoire Sols et Environnement, Université de Lorraine-INRAE, Vandœuvre-lés-Nancy, UMR 1120, France; Laboratory of Genetics, Wageningen University and Research, The Netherlands; Department of Botany and Zoology, Stellenbosch University, Matieland 7602, South Africa; Department of Botany and Zoology, Stellenbosch University, Matieland 7602, South Africa; AGH University of Science and Technology, Faculty of Physics & Applied Computer Science, 30-059 Kraków, Poland; Deutsches Elektronen-Synchrotron DESY, 22607 Hamburg, Germany; Department of Physics, Universität Hamburg, 20355 Hamburg, Germany; Faculty of Chemistry and Biochemistry, Ruhr-Universität Bochum, 44801 Bochum, Germany; Department of Chemistry, The University of Adelaide, Adelaide 5005, Australia

**Keywords:** hyperaccumulator, reconstructions, seed, synchrotron, X-ray fluorescence microscopy, X-ray fluorescence tomography

## Abstract

The molecular biology and genetics of the Ni–Cd–Zn hyperaccumulator *Noccaea caerulescens* has been extensively studied, but no information is yet available on Ni and Zn redistribution and mobilization during seed germination. Due to the different physiological functions of these elements, and their associated transporter pathways, we expected differential tissue distribution and different modes of translocation of Ni and Zn during germination. This study used synchrotron X-ray fluorescence tomography techniques as well as planar elemental X-ray imaging to elucidate elemental (re)distribution at various stages of the germination process in contrasting accessions of *N. caerulescens*. The results show that Ni and Zn are both located primarily in the cotyledons of the emerging seedlings and Ni is highest in the ultramafic accessions (up to 0.15 wt%), whereas Zn is highest in the calamine accession (up to 600 μg g^–1^). The distribution of Ni and Zn in seeds was very similar, and neither element was translocated during germination. The Fe maps were especially useful to obtain spatial reference within the seeds, as it clearly marked the vasculature. This study shows how a multimodal combination of synchrotron techniques can be used to obtain powerful insights about the metal distribution in physically intact seeds and seedlings.

## Graphical Abstract

**Graphical Abstract:**
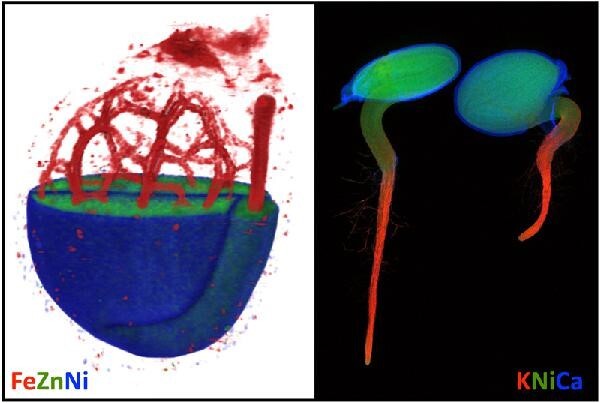
Tomographic reconstruction and 2D elemental mapping of *Noccaea caerulescens* seeds.

## Introduction

Hyperaccumulator plants are the perfect model systems for advancing our comprehension of the molecular biology and physiology of the regulation of transition elements, including Ni, Mn, and Zn.^1–3^ The localization of transition elements in hyperaccumulator seeds in relation to the germination processes remains understudied. Seed germination is a vital phase in the lifecycle of plants as the nascent seed has to contain all of the essential elements needed for emergence of the young plant.^4^ Previous studies on *Berkheya coddii* showed that Ni is located mainly in the micropylar area, in the embryo, and in the lower epidermis of cotyledons, whereupon it is mobilized to leaf palisade parenchyma after the first leaves emerge.^5^ In *Noccaea praecox*, Cd was translocated to the shoots (and not the roots) during germination.^6^

The genus *Noccaea* is unique in that it has over 20 taxa that hyperaccumulate different transition elements (Ni, Zn, Cd, and Pb) with several species hyperaccumulating more than one element simultaneously.^7–9^ The levels of efficiency and specificity can be remarkable, e.g. *Noccaea caerulescens* can accumulate 8890 μg g^–^^1^ Zn from a soil with only 139 μg g^–^^1^ Zn.^10^ This species is unique in that it possesses calamine, ultramafic, and non-metallicolous ecotypes, each represented by different accessions, which differ in tolerance and hyperaccumulation. All calamine, ultramafic, and non-metallicolous *N. caerulescens* accessions hyperaccumulate Zn and Ni, while not all will accumulate Cd when it is supplied in their environment.^11,12^*Noccaea caerulescens* accessions display remarkable differences in their tolerance of these metals depending on the origin.^13–15^ Not only is *N. caerulescens* of interest because of the spectrum of metals it tolerates and hyperaccumulates, it makes for a powerful model system because of the availability of a full molecular toolkit with the nuclear genome and transcriptome of this species having been established (Severing and Aarts, unpublished work).^16^ Consequently, candidate genes involved in Ni and, mainly, Zn uptake and translocation are now known.^17–24^ Zinc appears to be taken up by plasma-membrane located ZIP (Zinc-regulated, Iron-regulated transporter-like Protein) Zn-importing transporters and is then loaded into the xylem by plasma-membrane located HMA (Heavy Metal ATPase 4) Zn-exporting transporters for translocation to the shoot.^25^ This most likely requires additional ZIP proteins and the vacuolar loading P-type ATPase HMA3.^21^ In addition, genetically segregating accessions have been generated by crossing different accessions, which can be used to genetically characterize different metal-adaptation traits,^26–28^ and for three metallicolous and three non-metallicolous natural accessions, whole-genome resequencing data are generated (Wang, van den Heuvel, and Aarts, unpublished work) for population genomics purposes.^29^ Next to these non-metallicolous and calamine accessions, ultramafic accessions are known,^15^ e.g. many *N. caerulescens* accessions have been cultivated in genetically homogeneous lines by recurrent inbreeding (Schat and Aarts, unpublished work). The diversity in metal tolerance/hyperaccumulation is heritable and not dependent on each other (meaning that hyperaccumulation can be combined with hypersensitivity and consequently lethality upon metal exposure).^26,27,30^ The calamine (as well as some non-metallicolous) accessions have been studied to much greater extent than those from ultramafic soils. Most investigations have focused on the ultramafic accession from Monte Prinzera, in Italy,^11^ including proteomics studies aimed at correlating adaptation to protein expression^31^ and transcriptome studies.^22^ Nickel, as well as Zn, xylem loading in *N. caerulescens* is facilitated by histidine,^28,32^ although there is no evidence that this involves 1:1 HIS–Ni complexation and the related genes are not yet known.

Information on the distribution of transition elements and translocation during the germination process will shed light on the seed physiology of hyperaccumulator plants.^5^ Currently there is no information on the redistribution and the translocation of Ni and Zn during seed germination in *N. caerulescens*. Synchrotron-based X-ray fluorescence microscopy (XFM) imaging can be used for increasing our understanding of the fundamental processes involved with hyperaccumulation at different length-scales (whole plant scale down to the subcellular level) and with sufficient sensitivity for the measurement of the entire metallome of a plant.^33,34^ The ability to perform element mapping of live seedlings is contingent on the ultra-fast X-ray detection of the Maia detector system (with per-pixel dwell as low as 0.1 ms), which avoids radiation damage and associated sample degradation to the specimens.^35,36^ Most synchrotron experiments acquire ‘2D’ elemental maps of leaves or tissue cross-sections, but X-ray fluorescence micro-computed tomography (XFM-CT) enables reconstruction of ‘virtual cross-sections’ or 3D models of elemental data from a series of projection images.^33,37^ It enables ‘virtual sectioning’ of specimens, thereby entirely avoiding artefacts arising from destructive sample preparation. This method is primarily limited by self-absorption of escaping fluorescent X-rays (which is highly dependent on the X-ray energy, and hence, the element of interest). Therefore, it lends itself best to specimens that are relatively thin along the two axes perpendicular to the axis of rotation, which means that plant seeds are ideal test subjects for this approach. A pioneering study by Kim *et al.*^38^ applied XFM-CT to reveal Fe localization in the provascular system of intact *Arabidopsis* seeds.

Due to the different physiological functions of Ni and Zn, both essential nutrients that are toxic at high concentrations, but with the first required in much lower quantities than the second and with only partly overlapping associated transporter pathways,^13^ we expect differential distribution of Ni and Zn in the seeds of *N. caerulescens* and different modes of translocation during germination. This research aims to obtain information on the spatially resolved elemental distribution in the seeds and seedlings in distinct accessions of *N. caerulescens* contrasting in their Ni and Zn accumulation characteristics, ranging from high Ni with low Zn to low Ni with high Zn. We use synchrotron X-ray fluorescence tomography for the intact seeds and planar elemental imaging for the seedlings to elucidate elemental distribution at various stages of the germination process.

## Materials and methods

### Seed collection and culture conditions

The seeds were sourced from *N. caerulescens* growing in the native habitat in France and Spain from different accessions: two ultramafic accessions (Vosges in France and Cira in Spain) and a non-ultramafic (calamine) accession (‘Ganges’ near St Laurent le Minier in France). The ecology and soil chemistry of these accessions has been described in detail elsewhere.^10,14,15,28^ The seeds were germinated on moist [deionized water (DI)] Wettex (Vileda) at 20°C under fluorescent lights for 5 days until the cotyledons emerged and the root and hypocotyl were fully formed.

### Bulk chemical analysis of seeds

The elemental concentrations of the seeds were determined after microwave acid digestion of ∼100 mg of air-dried seeds using 4 ml HNO_3_ (70%) and subsequently analysed using inductively coupled plasma atomic emission spectroscopy (ICP–AES, Thermo Scientific iCAP 7400), as described earlier.^39^

### Scanning electron microscopy

**Fig. 1:**
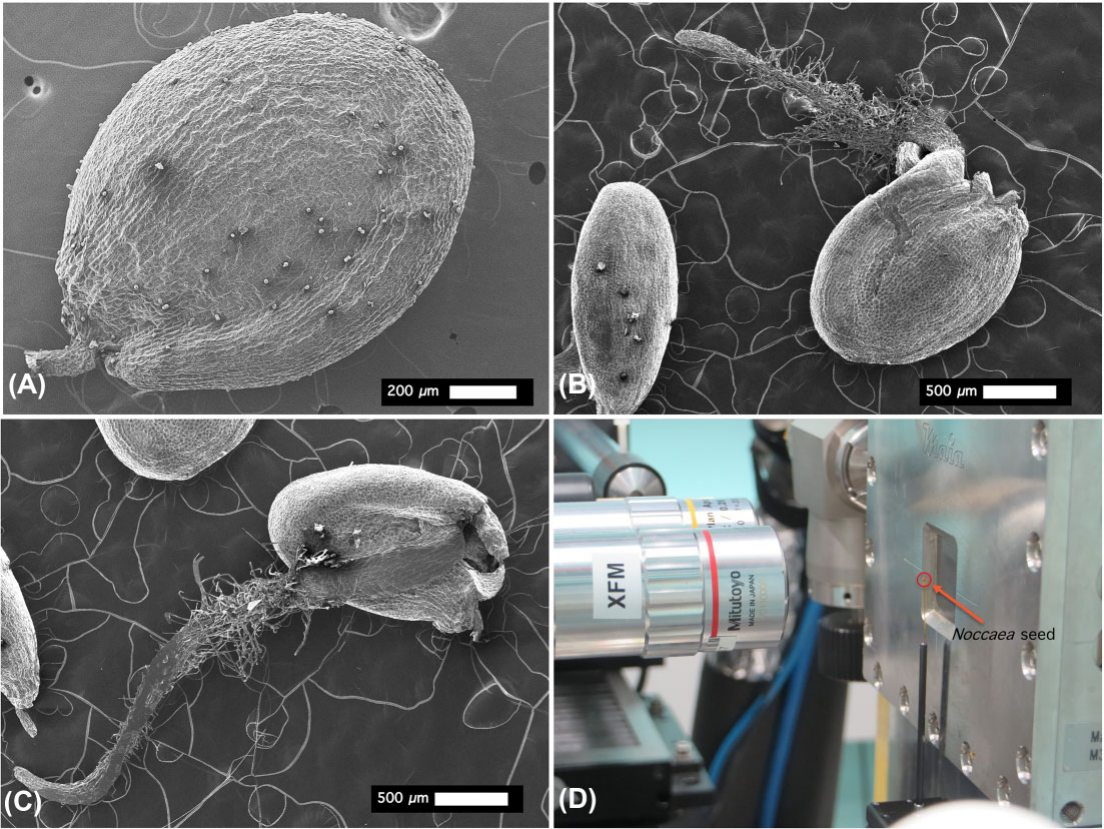
Scanning electron microscopy (SEM) images of intact *Noccaea caerulescens* seeds (panel **A**) and seedlings (panels **B** and **C**), and capillary-mounted seed on the tomographic stage in front of the Maia detector at the X-ray fluorescence microscopy (XFM) beamline (panel **D**).

The seeds were carbon coated and mounted on stubs and imaged with scanning electron microscopy (SEM) using a JEOL JSM-6610 instrument. Images were acquired at 100–200× magnification at 5 kV accelerator energy (Fig. 1A–C), as described earlier.^39^

### XFM experiments

The XFM beamline of the Australian Synchrotron produces monochromatic radiation in the range of 4.1–20 keV with a focus down to ∼1 μm^40^ and a Maia detector in backscatter geometry.^41,42^ The 2D elemental maps of the live seedlings were acquired using an incident energy of 15.8 keV, while the 3D tomograms of the seeds were acquired by collecting 2D XFM maps viewed between 152 and 251 angles spaced over 360°. The horizontal pixel size was either 5 microns with a dwell time of 1 ms, or 8 microns with a dwell time of 1.6 ms, while in the vertical direction the pixel size was twice that of the horizontal. In total, six seeds (two replicates from each of the three accessions Ganges, Vosges, and Cira) were imaged (Fig. 1D). The intact seeds were mounted, using a tiny amount of cyanoacrylate glue, on a needle attached to the tomography stage. The whole intact live seedlings were held between two sheets of polyethylene (Ultralene) thin film (4 μm) held over a Perspex frame in front of the Maia Detector. The two sheets of Ultralene thin film prevent dehydration during the measurement, which on average took just 30 min per sample. To avoid potential artefacts capable of confounding the collected data due to measurement conditions, we undertook fast scanning (to minimize the effective radiation dose) and applied a cold shock (by contacting the seedlings with ice cold water for 30 min immediately prior to the analysis) to slow down metabolic processes during the analysis and to reduce beam damage.^39^

### Data analysis

The X-ray Fluorescence Spectroscopy (XRF) event stream was analysed using the dynamic analysis method^43,44^ using the GeoPIXE package.^45,46^ The analysis of the tomographic data consisted of alignment and reconstruction steps. In case of the 3D tomograms the alignment steps were cross-correlated in the vertical direction and a combined consistency and cross-correlation method was applied in the horizontal direction. Considering that the single-slice tomograms lack vertical information, they were only aligned in horizontal direction. The aligned data were then reconstructed using a maximum-likelihood expectation–maximization algorithm based on the functions of the scikit-image Python library. Finally, the 3D renderings of the seeds were created using the open-source scientific visualization software Drishti.^47^

## Results

### Elemental concentrations in seeds

The seeds originating from the French accessions (Ganges, Vosges) and Spain (Cira) differed in their concentrations of Ni and Zn (Table 1). Seeds from ultramafic locations (Vosges, Cira) were characterized by very high concentrations of Ni (1000 and 1600 μg g^−1^ dry weight, respectively) whereas the seeds from the non-ultramafic (Ganges, Zn–Pb enriched ‘calamine’) location had only 65 μg g^−1^ Ni. The Zn concentration was highest in seeds from the Ganges accession (700 μg g^–1^ dry weight) while in the other two accessions it was only between 100 and 190 μg g^–1^ dry weight. Furthermore, an exceptionally high concentration of Cd was recorded in seeds from the Ganges location (704 μg g^–1^ dry weight) while in the other accession, Cd reached only a few micrograms per gram (μg g^–1^) dry weight. Other microelements (Mn, Fe, Co, Cu) were within the normal physiological range for plants.

**Table 1.** Elemental concentrations in dry seeds of *Noccaea caerulescens* originating from Cira (Spain) and Vosges and Ganges (France)^a^

Accession	Mg	P	K	Ca	Mn	Fe	Co	Ni	Cu	Zn	Cd	Pb
Cira	1190	5470	4680	3680	81	53	1.44	1600	2.99	102	4.27	1.65
Vosges	2950	6760	6000	3980	143	141	9.91	1000	2.89	191	3.12	1.37
Ganges	2250	6160	5500	7650	103	68	1.05	65	17	700	704	4.08

^a^All concentrations in micrograms per gram (μg g^–1^) dry weight.

### Tomographic reconstructions of intact non-germinated seeds

**Fig. 2:**
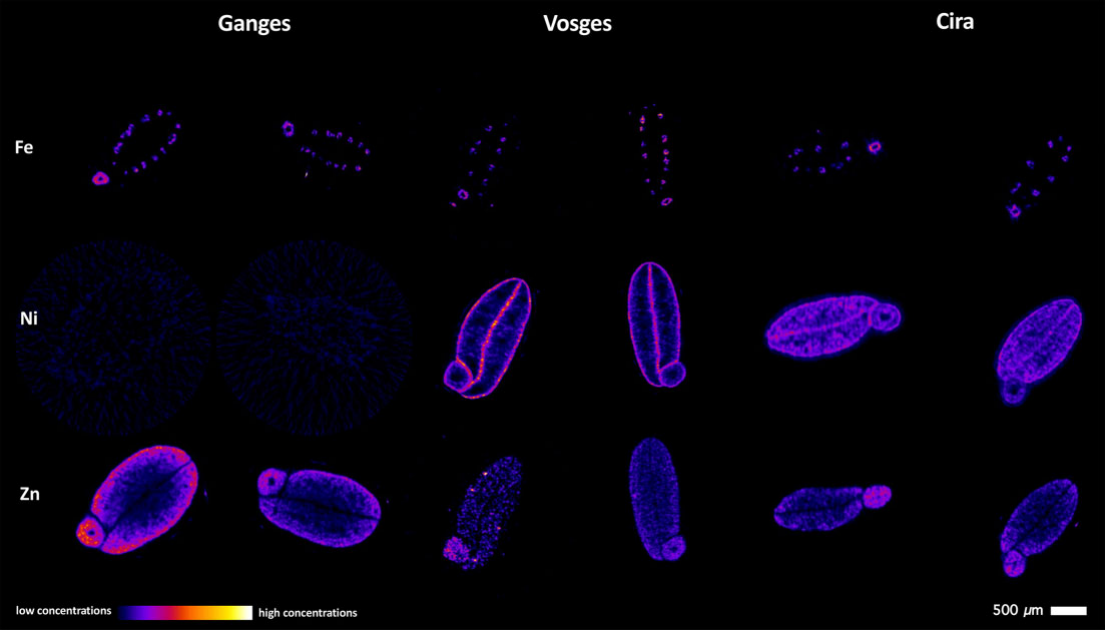
Single-slice X-ray fluorescence microscopy (XFM) tomograms of dry *Noccaea caerulescens* seeds showing Fe, Ni, and Zn signals within the physically intact seeds. The seed accessions shown here originate from Ganges (non-ultramafic, calamine) and Vosges (ultramafic) in France and Cira (ultramafic) in Spain.

**Fig. 3:**
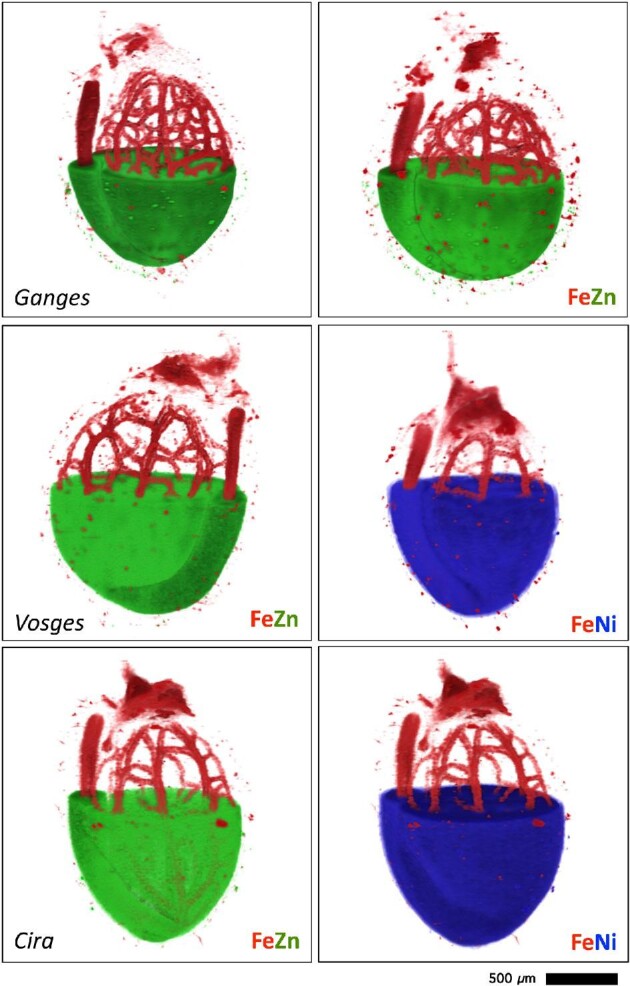
Tomographic reconstruction of dry *Noccaea caerulescens* seeds showing Fe and Ni or Zn signals within the physically intact seeds. The seed accessions shown here originate from Ganges (non-ultramafic, calamine) and Vosges (ultramafic) in France and Cira (ultramafic) in Spain.

The 3D tomography was undertaken in seeds from the Ganges, Vosges, and Cira accessions in duplicate (Fig. 2). For each seed ∼250 slices were obtained. Based on the slices, 3D tomographic reconstructions of dry *N. caerulescens* seeds were made (Fig. 3, Supplementary Fig. S1). The Fe maps are very similar between the accessions, with the characteristic provascular strand network of Fe enrichment in the radicle and vasculature of the cotyledons. Iron is also high in the endosperm and hilum area. Only the Vosges and Cira accessions (which are from ultramafic soils) have detectable Ni. Nickel concentrations are much higher in Vosges than in Cira seeds (although the concentrations from tomography are not quantitative, but can be gauged from raw intensity values), with Ni present in the epidermal cells of the cotyledons and radicle, and minor enrichment in the cotyledon blades (but notably not in the vascular bundles) (Fig. 2). This pattern is similar in the seeds of the Cira accessions, albeit less distinct due to the lower prevailing concentrations. The Zn concentrations are highest in Ganges (which is non-ultramafic, but calamine), lower in the Cira accession, and lowest in the Vosges accession; however, the Zn distribution patterns appear to be similar between all three accessions. Zinc concentrations are highest in the cortex of the radicle, and in the outer part of the cotyledon blades. Vascular bundles, including the central nerve, are depleted in Zn.

### Elemental distribution in live seedlings at different stages of development

**Fig. 4:**
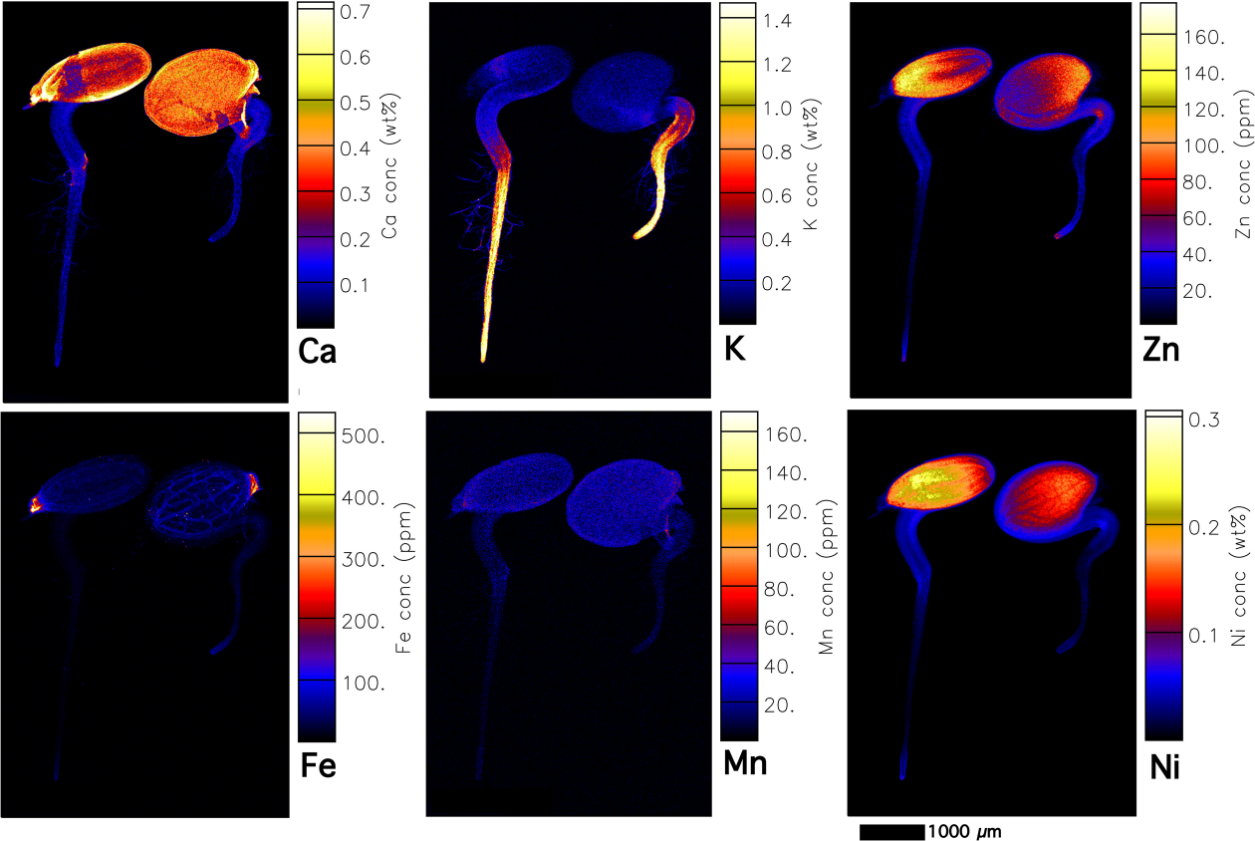
Elemental X-ray fluorescence microscopy (XFM) maps of living *Noccaea caerulescens* seedlings (Vosges accession, ultramafic). The scan size is 44 × 9.8 mm (885 × 1240 pixels). The elemental image was acquired in 5 μm step size with 2.5 ms dwell per pixel, 15.8 keV, incident beam, showing K, Ca, Fe, Mn, Ni, and Zn maps.

**Fig. 5:**
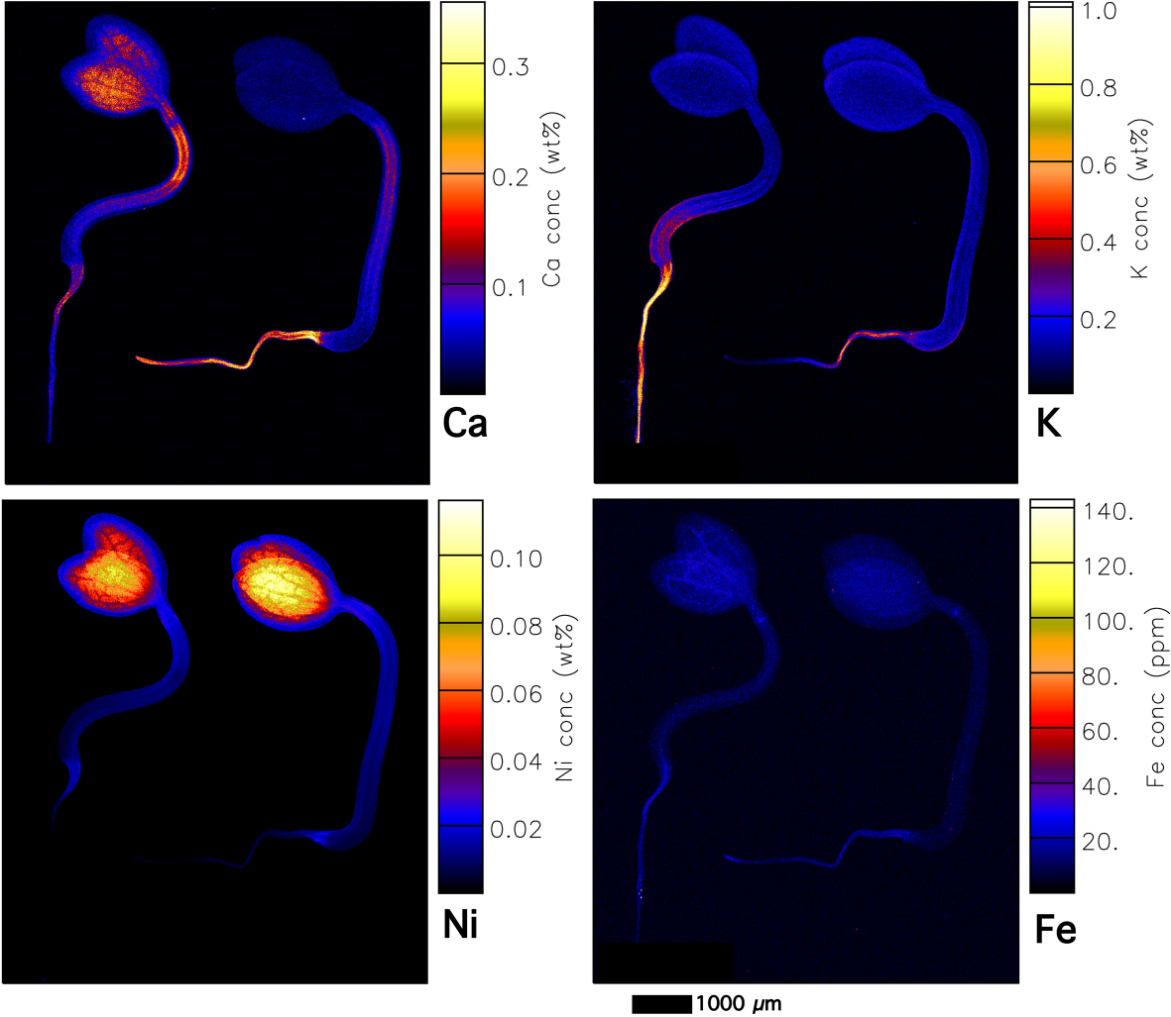
Elemental X-ray fluorescence microscopy (XFM) maps of living *Noccaea caerulescens* seedlings (Cira accession, ultramafic). The scan size is 21.5 × 89 mm (1448 × 1500 pixels). The elemental image was acquired in 5 μm step size with 1.7 ms dwell per pixel, 15.8 keV, incident beam, showing K, Ca, Fe, and Ni maps.

**Fig. 6:**
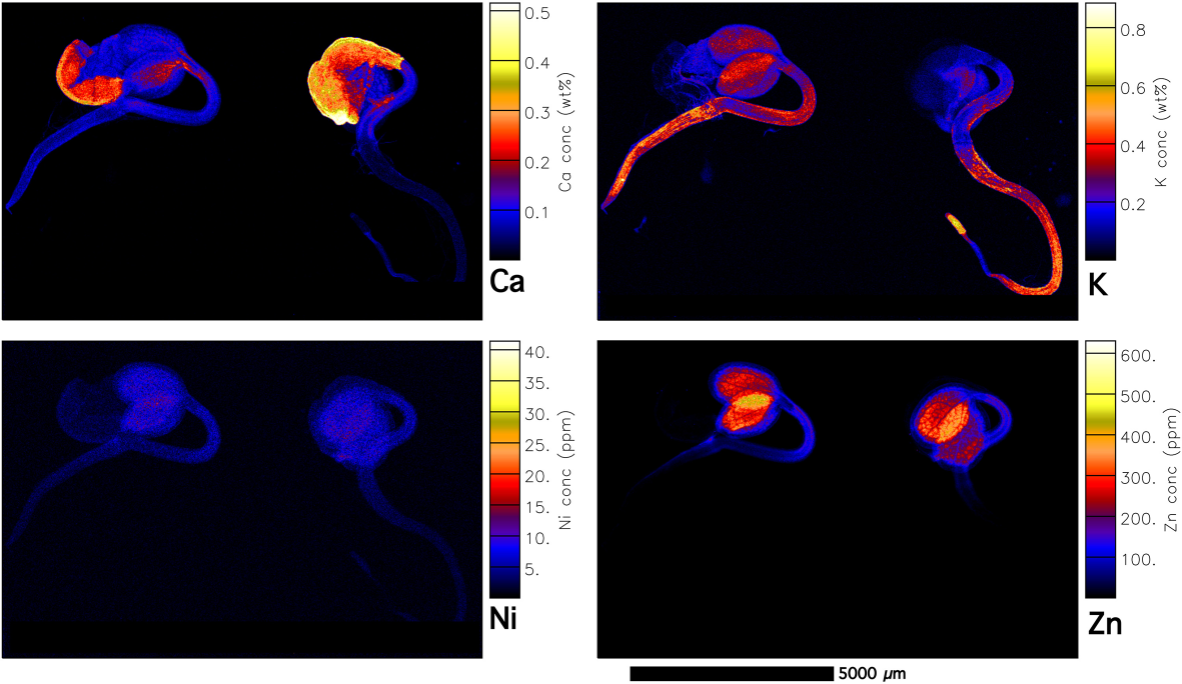
Elemental X-ray fluorescence microscopy (XFM) maps of living *Noccaea caerulescens* seedlings (Ganges accession, non-ultramafic, calamine). The scan size is 21.8 × 31 mm (2366 × 1562 pixels). The elemental image was acquired in 5 μm step size with 2.5 ms dwell per pixel, 15.8 keV, incident beam, showing K, Ca, Ni, and Zn maps.

In all accessions, Fe is particularly enriched in the cotyledon provascular bundles as well as in the hypocotyl (Figs 4 and 5, Supplementary Figs S3 and S6). Iron concentrations locally reach up to 500 μg g^–1^ and are highest in hotspots in the hilum of the micropylar area (Fig. 4). Iron enrichment in the provascular strands disappears when the seedling develops further. Manganese is distributed throughout the seeds (mainly cotyledons), and lower in the root (Fig. 4). The distribution of Ni and Zn is very similar in most accessions and strongly enriched in the cotyledons. Whereas Ni is especially high (up to 0.25 wt%) in the central part of the cotyledon (Fig. 4, Supplementary Fig. S3), Zn is enriched toward the apex of the cotyledon blade (up to 400 μg g^–1^). In the calamine accession Zn reaches up to 400 μg g^–1^ (Fig. 6), and the distribution pattern is similar in the ultramafic accessions (Vosges, France, and Circa, Spain, which have even higher Zn enrichment, up to 600 μg g^–1^; Supplementary Fig. S7). In the young seedlings from ultramafic accessions, Zn is evenly distributed through the cotyledon blade, with an increase toward the apex (Supplementary Fig. S7). In the calamine accession Zn is highest in the blade margins (Supplementary Figs S4 and S6).

During the first stage of germination (e.g. emergence of the cotyledons and radicle), K is strongly enriched in the radicle (especially in the root apex) and hypocotyl (Fig. 6) and this pattern remains during further development in seedlings from both ultramafic and non-ultramafic accessions (Figs 4 and 5, Supplementary Figs S2–7). In later stages of development (e.g. full extension of the hypocotyl and root), K is enriched in the radicle and can reach up to 2 wt% (Supplementary Fig. S4), before it is translocated into the hypocotyl. Comparing seedlings in different stages of development (compare K maps of seedlings with (Fig. 2) and without dehisced seed coat (Figs 3 and 5), it appears that K is translocated to the radicle from the cotyledons during seedlings’ emergence. Calcium is relatively low in the seedlings (<0.1 wt%), but strongly enriched in the dehisced seed coat with up to 0.5 wt% Ca (Fig. 6; Supplementary Figs S2 and S7). In more developed seedlings from both ultramafic and non-ultramafic accessions (Fig. 5), Ca is enriched in the epicotyl and hypocotyl (especially in the node between epicotyl and hypocotyl).

## Discussion

Synchrotron XFM-CT enables ‘virtual sectioning’ of a specimen thereby entirely avoiding artefacts arising from destructive sample preparation, beyond removal from the native environment. As such, it has broad utility across the biological and medical sciences, but this is mostly yet to be realized. Herein, topographical reconstructions of intact seeds from metal hyperaccumulating plants revealed distinct localization of Ni and Zn in the anatomical structures of the seeds, showcasing the usefulness of this analytical approach.

This study sought to investigate the distribution of Ni and Zn in intact seeds of the Ni–Cd–Zn hyperaccumulator *N. caerulescens* using XFM techniques, and the distribution of these and other physiologically relevant elements during seed germination. We used seeds from different accessions contrasting in their Ni and Zn hyperaccumulation characteristics (ultramafic versus calamine accessions). All accessions of *N. caerulescens* hyperaccumulate Zn, regardless of the nature of the soil. Nickel hyperaccumulation, however, is limited to ultramafic soils in the field, though different accessions originating from calamine or non-metallicolous soil will be able to take up Ni at high levels above the hyperaccumulation threshold as well. While we expected differential tissue distribution and different modes of translocation of Ni and Zn during germination, this is not the case. Nickel and Zn are both located primarily in the cotyledons of the emerging seedlings and Ni is highest in the ultramafic accessions (0.16 wt% on the basis of ICP–AES data), whereas Zn is highest in the calamine accession (700 μg g^–1^ on the basis of ICP–AES data). However, the Zn concentrations in the seeds are surprisingly low and on parity with Cd concentrations in some of the seeds (with 700 μg g^–1^ on the basis of ICP–AES data of the Ganges accession) or compared to Ni concentrations in the seeds from ultramafic accessions. This aligns with a study on *N. praecox* in which it was found that Cd was accumulated at higher concentrations in the seeds than Zn, while in the leaves Zn reaches 10 times the concentrations of Cd.^6^ The distribution of Ni and Zn in seeds was very similar, and neither element was translocated during germination. Zinc was highest in the interveinal areas of the cotyledons. The Fe maps were especially useful to obtain spatial reference within the seeds, as it clearly marked the provascular strands. Hence, the Fe distribution pattern, visible as a network of provascular strands in the hypocotyl, radicle, and cotyledons, as first reported in *Silene vulgaris*^48^ and later in other species, including *Arabidopsis thaliana*,^38,49^*Arabidopsis halleri*,^50^ and *Biscutella laevigata*,^51^ is also present in *N. caerulescens*. Nickel and Zn were enriched in the epidermal cells of the cotyledons increasing toward the cotyledon margins, and not in the mesophyll cells that surround the provascular strands. This strongly suggests that Ni and Zn are co-located in the epidermal cells (likely the vacuoles), as in mature plants.

In the seeds of *Noccaea pindica*, Ni is strongly enriched in the micropylar area near the radicle as well as in the cotyledon epidermis.^52^ In other hyperaccumulator plants, the cotyledons (in *Hybanthus floribundus* subsp. *adpressus*—Violaceae), the embryonic axis (in *Pimelea leptospermoides*—Thymelaeaceae), and the pericarp (in *Stackhousia tryonii*—Celastraceae) were the main enrichment areas for Ni.^53,54^ In *Berkheya coddii* (Asteraceae), Ni was in the micropylar area, at the bottom of the embryo, in the lower epidermis and the margins of the cotyledons. Nickel was localized largely in the cotyledons and translocated to the leaves after emergence, where Ni was accumulated in the leaf margins and in the leaf midrib.^5^ In *Odontarrhena corsica* (synonym *Alyssum corsicum*) and *Odontarrhena chalcidica* (synonym *Alyssum murale*) (both Brassicaceae), Ni predominately accumulated in the cotyledons as well as the hypocotyl, but it was comparatively low in the seed coat.^55^ Thus, it seems that the results we report here for *N. caerulescens* align very well with what has been found for other, related, and non-related, hyperaccumulator species, with respect to localization of Ni and Zn in the epidermal cells of the cotyledons.

## Conclusions

Most investigations employ X-ray microanalysis (SEM–energy-dispersive X-ray spectroscopy (EDS), micro-PIXE, XFM) on hyperaccumulator plants to obtain elemental distribution data to infer fundamental molecular mechanisms *a posteriori* (including the present study). Such approaches have yielded a wealth of knowledge about the ecophysiology of hyperaccumulator plants. X-ray microanalysis can also be used to elicit far more insightful information when employed on plant models *a priori* using mutants, including those in which genes encoding for metal transport proteins have been knocked out.^38,56,57^ The model species *N. caerulescens* is perfect for these lines of inquiry as it is a polymetallic (Cd–Ni–Zn–Pb) hyperaccumulator for which there is a molecular toolkit available to study and manipulate gene expression.^12,16^

## Author contributions

A.vdE. and G.E. collected the seeds in the field. A.vdE. prepared the samples for the synchrotron analysis. A.vdE., M.D.dJ., J.M-P., W.J.P., and H.H.H. conducted the synchrotron XFM experiments at the Australian Synchrotron. A.vdE. performed the SEM and light microscopy experiments. D.B. reconstructed the tomograms and created the 3D renderings. All authors contributed to writing the manuscript.

## Supplementary Material

mfac026_Supplemental_Files

## Data Availability

The data underlying this article will be shared on reasonable request to the corresponding author.

## References

[1] Pollard A. J. , Powell K. D., Harper F. A., Smith J. A. C., The Genetic Basis of Metal Hyperaccumulation in Plants, Crit. Rev. Plant Sci., 2002, 21, 539–566.

[2] Krämer U. , Metal Hyperaccumulation in Plants, Annu. Rev. Plant Biol., 2010, 61, 517–534. 20192749 10.1146/annurev-arplant-042809-112156

[3] Van Der Ent A. , Baker A. J. M., Reeves R. D., Pollard A. J., Schat H., Hyperaccumulators of Metal and Metalloid Trace Elements: Facts and Fiction, Plant Soil, 2013, 362, 319–334.

[4] Schnell Ramos M. , Khodja H., Mary V., Thomine S., Using μPIXE for Quantitative Mapping of Metal Concentration in *Arabidopsis thaliana* Seeds, Front. Plant Sci., 2013, 4. doi: 10.3389/fpls.2013.00168. 10.3389/fpls.2013.00168 PMC3669754 23761799

[5] Groeber S. , Przybyłowicz W., Echevarria G., Montarges-Pelletier E., Barnabas A., Mesjasz-Przybyłowicz J., Fate of Nickel and Calcium in Seedlings of the Hyperaccumulator *Berkheya coddii* during Germination, Biologia Plantarum, 2015, 59, 560–569.

[6] Vogel-Mikuš K. , Pongrac P., Kump P., Nečemer M., Simčič J., Pelicon Pž, Budnar M., Povh B., Regvar M., Localisation and Quantification of Elements within Seeds of Cd/Zn Hyperaccumulator *Thlaspi praecox* by Micro-PIXE, Environ. Pollut., 2007, 147, 50–59. 17070633 10.1016/j.envpol.2006.08.026

[7] Reeves R. D. , Brooks R. R., Hyperaccumulation of Lead and Zinc by Two Metallophytes from Mining Areas of Central-Europe, Environmental Pollution Series A-Ecological and Biological, 1983, 31, 277–285.

[8] Lombi E. , Zhao F. J., Dunham S. J., Mcgrath S. P., Cadmium Accumulation in Populations of *Thlaspi caerulescens and Thlaspi goesingense*, New Phytol., 2000, 145, 11–20.

[9] Vogel-Mikuš K. , Drobne D., Regvar M., Zn, Cd and Pb Accumulation and Arbuscular Mycorrhizal Colonisation of Penny-Cress *Thlaspi praecox* Wulf. (Brassicaceae) from the Vicinity of a Lead Mine and Smelter in Slovenia, Environ. Pollut., 2005, 133, 233–242. 15519454 10.1016/j.envpol.2004.06.021

[10] Reeves R. D. , Schwartz C., Morel J. L., Edmondson J., Distribution and Metal-Accumulating Behavior of *Thlaspi caerulescens* and Associated Metallophytes in France, Int. J. Phytorem., 2001, 3, 145–172.

[11] Assunção A. G. L. , Bookum W. M., Nelissen H. J. M., Vooijs R., Schat H., Ernst W. H. O., Differential Metal-Specific Tolerance and Accumulation Patterns among *Thlaspi caerulescens* Populations Originating from Different Soil Types, New Phytol., 2003, 159, 411–419. 33873347 10.1046/j.1469-8137.2003.00819.x

[12] Assunção A. G. L. , Schat H., Aarts M. G. M., *Thlaspi caerulescens*, an Attractive Model Species to Study Heavy Metal Hyperaccumulation in Plants, New Phytol., 2003, 159, 351–360. 33873356 10.1046/j.1469-8137.2003.00820.x

[13] Deng T.-.H.-.B. , Cloquet C., Tang Y.-.T., Sterckeman T., Echevarria G., Estrade N., Morel J.-L., Qiu R.-L., Nickel and zinc Isotope Fractionation in Hyperaccumulating and Nonaccumulating Plants, Environ. Sci. Technol., 2014, 48, 11926–11933. 25222693 10.1021/es5020955

[14] Gonneau C. , Genevois N., Frérot H., Sirguey C., Sterckeman T., Variation of Trace Metal Accumulation, Major Nutrient Uptake and Growth Parameters and Their Correlations in 22 Populations of *Noccaea caerulescens*, Plant Soil, 2014, 384, 271–287.

[15] Gonneau C. , Noret N., Godé C., Frérot H., Sirguey C., Sterckeman T., Pauwels M., Demographic History of the Trace Metal Hyperaccumulator *Noccaea caerulescens* (J. Presl and C. Presl) F. K. Mey. in Western Europe, Mol. Ecol., 2017, 26 (3), 904–922. 27914207 10.1111/mec.13942

[16] Lin Ya-F , Severing E. I., Te Lintel Hekkert B., Schijlen E., Aarts M. G. M.., A Comprehensive Set of Transcript Sequences of the Heavy Metal Hyperaccumulator *Noccaea caerulescens*, Front. Plant Sci., 2014, 5, 261. 24999345 10.3389/fpls.2014.00261 PMC4064536

[17] Assunção A. G. L. , Martins P. D. C., De Folter S. et al., Elevated Expression of Metal Transporter Genes in Three Accessions of the Metal Hyperaccumulator *Thlaspi caerulescens*, Plant, Cell & Environment, 2001, 24, 217–226.

[18] Oomen R. , Wu J., Lelièvre F., Blanchet S., Richaud P., Barbier-Brygoo H., Aarts M. G. M., Thomine S., Functional Characterization of NRAMP3 and NRAMP4 from the Metal Hyperaccumulator *Thlaspi caerulescens*, New Phytol., 2009, 181, 637–650. 19054339 10.1111/j.1469-8137.2008.02694.x

[19] Wu J. , F.-J., Zhao., Ghandilyan A., Logoteta B., Guzman M. O., Schat H., Wang X., Aarts M. G. M., Identification and Functional Analysis of Two ZIP Metal Transporters of the Hyperaccumulator *Thlaspi caerulescens*, Plant Soil, 2009, 325, 79–95.

[20] Ó Lochlainn S. , Bowen H. C., Fray R. G., Hammond J. P., King G. J., White P. J., Graham N. S., Broadley M. R., Tandem Quadruplication of *HMA4* in the Zinc (Zn) and Cadmium (Cd) Hyperaccumulator *Noccaea caerulescens* (I Baxter, Ed.), PLoS One, 2011, 6, e17814–e17819. 21423774 10.1371/journal.pone.0017814 PMC3053397

[21] Ueno D. , Milner M. J., Yamaji N., Yokosho K., Koyama E., Clemencia Zambrano M., Kaskie M., Ebbs S., Kochian L. V., Ma J. F., Elevated Expression of TcHMA3 Plays a Key Role in the Extreme Cd Tolerance in a Cd-Hyperaccumulating Ecotype of *Thlaspi caerulescens*, Plant J., 2011, 66, 852–862. 21457363 10.1111/j.1365-313X.2011.04548.x

[22] Halimaa P. , Lin Y.-F., Ahonen V. H. et al., Gene Expression Differences between *Noccaea caerulescens* Ecotypes Help to Identify Candidate Genes for Metal Phytoremediation, Environ. Sci. Technol., 2014, 48, 3344–3353. 24559272 10.1021/es4042995

[23] Lin Ya-F , Hassan Z., Talukdar S., Schat H., Aarts M. G. M., Expression of the ZNT1 Zinc Transporter from the Metal Hyperaccumulator *Noccaea caerulescens* Confers Enhanced Zinc and Cadmium Tolerance and Accumulation to *Arabidopsis thaliana* (I Baxter, Ed.), PLoS One, 2016, 11, e0149750–30. 26930473 10.1371/journal.pone.0149750 PMC4773103

[24] Blande D. , Halimaa P., Tervahauta A. I., Aarts M. G. M., Kärenlampi S. O., De Novo Transcriptome Assemblies of Four Accessions of the Metal Hyperaccumulator Plant *Noccaea caerulescens*, Scientific Data, 2017, 4, 160131. 28140388 10.1038/sdata.2016.131 PMC5283065

[25] Hanikenne M. , Talke I. N., Haydon M. J., Lanz C., Nolte A., Motte P., Kroymann J., Weigel D., Krämer U., Evolution of Metal Hyperaccumulation Required *Cis*-Regulatory Changes and Triplication of *HMA4*, Nature, 2008, 453, 391–395. 18425111 10.1038/nature06877

[26] Assuncao A. G. L. , Pieper B., Vromans J., Lindhout P., Aarts M. G. M., Schat H., Construction of a Genetic Linkage Map of *Thlaspi caerulescens* and Quantitative Trait Loci Analysis of Zinc Accumulation, New Phytol., 2006, 170, 21–32. 16539600 10.1111/j.1469-8137.2005.01631.x

[27] Deniau A. X. , Pieper B., Ten Bookum W. M., Lindhout P., Aarts M. G. M., Schat H., QTL Analysis of Cadmium and Zinc Accumulation in the Heavy Metal Hyperaccumulator *Thlaspi caerulescens*, Theor. Appl. Genet., 2006, 113, 907–920. 16850314 10.1007/s00122-006-0350-y

[28] Richau K. H. , Schat H., Intraspecific Variation of Nickel and Zinc Accumulation and Tolerance in the Hyperaccumulator *Thlaspi caerulescens*, Plant Soil, 2009, 314, 253–262.

[29] Turner T. L. , Bourne E. C., Von Wettberg E. J., Hu T. T., Nuzhdin S. V., Population Resequencing Reveals Local Adaptation of *Arabidopsis lyrata* to Serpentine Soils, Nat. Genet., 2010, 42, 260–263. 20101244 10.1038/ng.515

[30] Xing J. P. , Jiang R. F., Ueno D., Ma J. F., Schat H., Mcgrath S. P., Zhao F. J., Variation in Root-to-Shoot Translocation of Cadmium and Zinc among Different Accessions of the Hyperaccumulators *Thlaspi caerulescens* and *Thlaspi praecox*, New Phytol., 2008, 178, 315–325. 18266619 10.1111/j.1469-8137.2008.02376.x

[31] Visioli G. , Vincenzi S., Marmiroli M., Marmiroli N., Correlation between Phenotype and Proteome in the Ni Hyperaccumulator *Noccaea caerulescens* Subsp. *caerulescens*, Environ. Exp. Bot., 2012, 77, 156–164.

[32] Kozhevnikova A. D. , Seregin I. V., Erlikh N. T., Shevyreva T. A., Andreev I. M., Verweij R., Schat H., Histidine-Mediated Xylem Loading of Zinc is a Species-Wide Character in *Noccaea caerulescens*, New Phytol., 2014, 203, 508–519. 24750120 10.1111/nph.12816

[33] van der Ent A. , Przybyłowicz W. J., de Jonge M. D. et al., X-ray Elemental Mapping Techniques for Elucidating the Ecophysiology of Hyperaccumulator Plants, New Phytol., 2018, 218, 432–452. 28994153 10.1111/nph.14810

[34] Kopittke P. M. , Punshon T., Paterson D. J., Tappero R. V., Wang P., Blamey F. P. C., Van Der Ent A., Lombi E., Synchrotron-Based X-ray Fluorescence Microscopy as a Technique for Imaging of Elements in Plants, Plant Physiol., 2018, 178, 507–523. 30108140 10.1104/pp.18.00759 PMC6181034

[35] Blamey F. P. C. , McKenna B. A., Li C. et al., Manganese Distribution and Speciation Help to Explain the Effects of Silicate and Phosphate on Manganese Toxicity in Four Crop Species, New Phytol., 2017, 96, 37–15. 10.1111/nph.14878 29091286

[36] Blamey F. P. C. , Paterson D. J., Walsh A., Afshar N., Mckenna B. A., Cheng M., Tang C., Horst W. J., Menzies N. W., Kopittke P. M., Time-Resolved X-ray fluorescence Analysis of Element Distribution and Concentration in Living Plants: an Example Using Manganese Toxicity in Cowpea Leaves, Environ. Exp. Bot., 2018, 156, 151–160.

[37] De Jonge M. D. , Vogt S., Hard X-Ray Fluorescence Tomography—an Emerging Tool For Structural Visualization, Curr. Opin. Struct. Biol., 2010, 20, 606–614. 20934872 10.1016/j.sbi.2010.09.002

[38] Kim S. A. , Punshon T., Lanzirotti A., Li L., Alonso JoséM, Ecker J. R., Kaplan J., Guerinot M. L., Localization of Iron in Arabidopsis Seed Requires the Vacuolar Membrane Transporter VIT1, Science, 2006, 314, 1295–1298. 17082420 10.1126/science.1132563

[39] Paul A. L. D. , Harris H. H., Erskine P. D., Przybyłowicz W., Mesjasz-Przybyłowicz J., Echevarria G., Van Der Ent A., Synchrotron μXRF Imaging of Live Seedlings of *Berkheya coddii* and *Odontarrhena muralis* during Germination and Seedling Growth, Plant Soil, 2020, 453 (1), 487–501.

[40] Paterson D. J. , De Jonge M. D., Howard D. L. et al., The X-Ray Fluorescence Microscopy Beamline at the Australian Synchrotron, AIP Conf. Proc., 2011, 1365, 219.

[41] Kirkham R. , Dunn P. A., Kuczewski A. J., The Maia Spectroscopy Detector System: Engineering for Integrated Pulse Capture, Low-Latency Scanning and Real-Time Processing, AIP Conference Proceedings 1234, 2010, 1234, 240–243. doi: 10.1063/1.3463181.

[42] Siddons D. P. , Kirkham R., Ryan C. G. et al. 2014., Maia X-ray Microprobe Detector Array System, J. Phys. Conf. Ser., 2010, 499, 012001–012010.

[43] Ryan C. G. , Jamieson D. N., Dynamic Analysis: On-Line Quantitative PIXE Microanalysis and Its Use in Overlap-Resolved Elemental Mapping, Nucl. Instrum. Methods Phys. Res., Sect. B, 1993, 77, 203–214.

[44] Ryan C. G. , Quantitative Trace Element Imaging Using PIXE and the Nuclear Microprobe, Int. J. Imaging Syst. Technol., 2000, 11 (4), 219–230.

[45] Ryan C. G. , Cousens D. R., Sie S. H., Griffin W. L., Quantitative Analysis of PIXE Spectra in Geoscience Applications, Nucl. Instrum. Methods Phys. Res., Sect. B, 1990, 49, 271–276.

[46] Ryan C. G. , Etschmann B. E., Vogt S., Maser J., Harland C. L., Van Achterbergh E., Legnini D., Nuclear Microprobe–Synchrotron Synergy: Towards Integrated Quantitative Real-Time Elemental Imaging Using PIXE and SXRF, Nucl. Instrum. Methods Phys. Res., Sect. B, 2005, 231, 183–188.

[47] Limaye A. , Drishti: a Volume Exploration and Presentation Toolset, SPIE 8506, Developments in X-Ray Tomography VIII, 85060X

[48] Mesjasz-Przybyłowicz J. , Grodzińska K., Przybyłowicz W. J., Godzik B., Szarek-Łukaszewska G. 1999., Micro-PIXE Studies of Elemental Distribution in Seeds of *Silene vulgaris* from a Zinc Dump in Olkusz, Southern Poland, Nucl. Instrum. Methods Phys. Res., Sect. B, 2012, 158, 306–311.

[49] Eroglu S. , Giehl R. F. H., Meier B., Takahashi M., Terada Y., Ignatyev K., Andresen E., Küpper H., Peiter E., Von Wirén N., Metal Tolerance Protein 8 Mediates Manganese Homeostasis and Iron Reallocation During Seed Development and Germination, Plant Physiol., 2017, 174, 1633–1647. 28461400 10.1104/pp.16.01646 PMC5490884

[50] Babst-Kostecka A. , Przybyłowicz W. J., Seget B., Mesjasz-Przybyłowicz J., Zinc Allocation to and within *Arabidopsis halleri* Seeds: Different Strategies of Metal Homeostasis in Accessions under Divergent Selection Pressure, Plant-Environ. Interact., 2020, 1, 207–220. 10.1002/pei3.10032 PMC10168052 37284210

[51] Babst-Kostecka A. , Przybyłowicz W. J., Van Der Ent A., Ryan C., Dietrich C. C., Mesjasz-Przybyłowicz J., Endosperm Prevents Toxic Amounts of Zn from Accumulating in the Seed Embryo—an Adaptation to Metalliferous Sites in Metal-Tolerant *Biscutella laevigata*, Metallomics, 2020, 12 (1), 42–53. 31720657 10.1039/c9mt00239a

[52] Psaras G. , Nickel Localization in Seeds of the Metal Hyperaccumulator *Thlaspi pindicum* Hausskn, Ann. Bot., 2001, 88, 513–516.

[53] Bhatia N. P. , Orlic I., Siegele R., Ashwath N., Baker A. J. M., Walsh K. B., Elemental Mapping Using PIXE Shows the Main Pathway of Nickel Movement Is Principally Symplastic within the Fruit of the Hyperaccumulator *Stackhousia tryonii*, New Phytol., 2003, 160, 479–488. 33873657 10.1046/j.1469-8137.2003.00912.x

[54] Kachenko A. G. , Bhatia N. P., Siegele R. et al., Nickel, Zn and Cd Localisation in Seeds of Metal Hyperaccumulators Using μ-PIXE Spectroscopy, Nucl. Instrum. Methods Phys. Res., Sect. B, 2009, 267, 12–13.

[55] Centofanti T. , Tappero R. V., Davis A. P., Chaney R. L., Chelator-Buffered Nutrient Solution is Ineffective in Extracting Ni from Seeds of *Alyssum*, Int. J. Phytorem., 2011, 13 (5), 434–440. 10.1080/15226514.2010.483264 21598774

[56] Punshon T. , Guerinot M. L., Lanzirotti A., Using Synchrotron X-Ray Fluorescence Microprobes in the Study of Metal Homeostasis in Plants, Ann. Bot., 2008, 103, 665–672. 10.1093/aob/mcn264 PMC2707871 19182222

[57] Punshon T. , Ricachenevsky F. K., Hindt M. N., Socha A. L., Zuber H., Methodological Approaches for Using Synchrotron X-Ray Fluorescence (SXRF) Imaging as a Tool in Ionomics: Examples from *Arabidopsis thaliana*, Metallomics, 2013, 5, 1133–1113. 23912758 10.1039/c3mt00120b PMC3869573

