# Supplementary material for: Multimodal synchrotron X-ray fluorescence imaging reveals elemental distribution in seeds and seedlings of the Zn–Cd–Ni hyperaccumulator Noccaea caerulescens
Source: Metallomics. 2022 Jun 23;14(5):mfac026. doi: 10.1093/mtomcs/mfac026 (PMC9226517; doi:10.1093/mtomcs/mfac026)
Supplement: mfac026_Supplemental_Files [file mfac026_supplemental_files.zip › Suppl_Info_Supplementary Material.pdf]

SUPPLEMENTARY INFORMATION

Multi-modal synchrotron X-ray fluorescence imaging reveals elemental distribution in seeds and seedlings of the Zn-Cd-Ni hyperaccumulator *Noccaea caerulescens*

Antony van der Ent[1], Martin D. de Jonge[2], Guillaume Echevarria[3], Mark G. M. Aarts[4], Jolanta Mesjasz-Przybyłowicz[5], Wojciech J. Przybyłowicz[5,6], Dennis Brueckner[7,8,9], Hugh H. Harris[10]

[1]Centre for Mined Land Rehabilitation, Sustainable Minerals Institute, The University of Queensland, Australia.

[2]Australian Synchrotron, X-ray Fluorescence Microscopy, ANSTO, Australia.

[3]Laboratoire Sols et Environnement, Université de Lorraine-INRAE, Vandœuvre-lès-Nancy, UMR 1120, France.

[4]Laboratory of Genetics, Wageningen University and Research, The Netherlands.

[5]Department of Botany and Zoology, Stellenbosch University, South Africa.

[6]AGH University of Science and Technology, Faculty of Physics & Applied Computer Science, Poland.

[7]Photon Science, Deutsches Elektronen-Synchrotron DESY, Germany.

[8]Department of Physics, University of Hamburg, Germany.

[9]Faculty of Chemistry and Biochemistry, Ruhr-University Bochum, Germany.

[10]Department of Chemistry, The University of Adelaide, Australia.

Corresponding author: a.vanderent@uq.edu.au

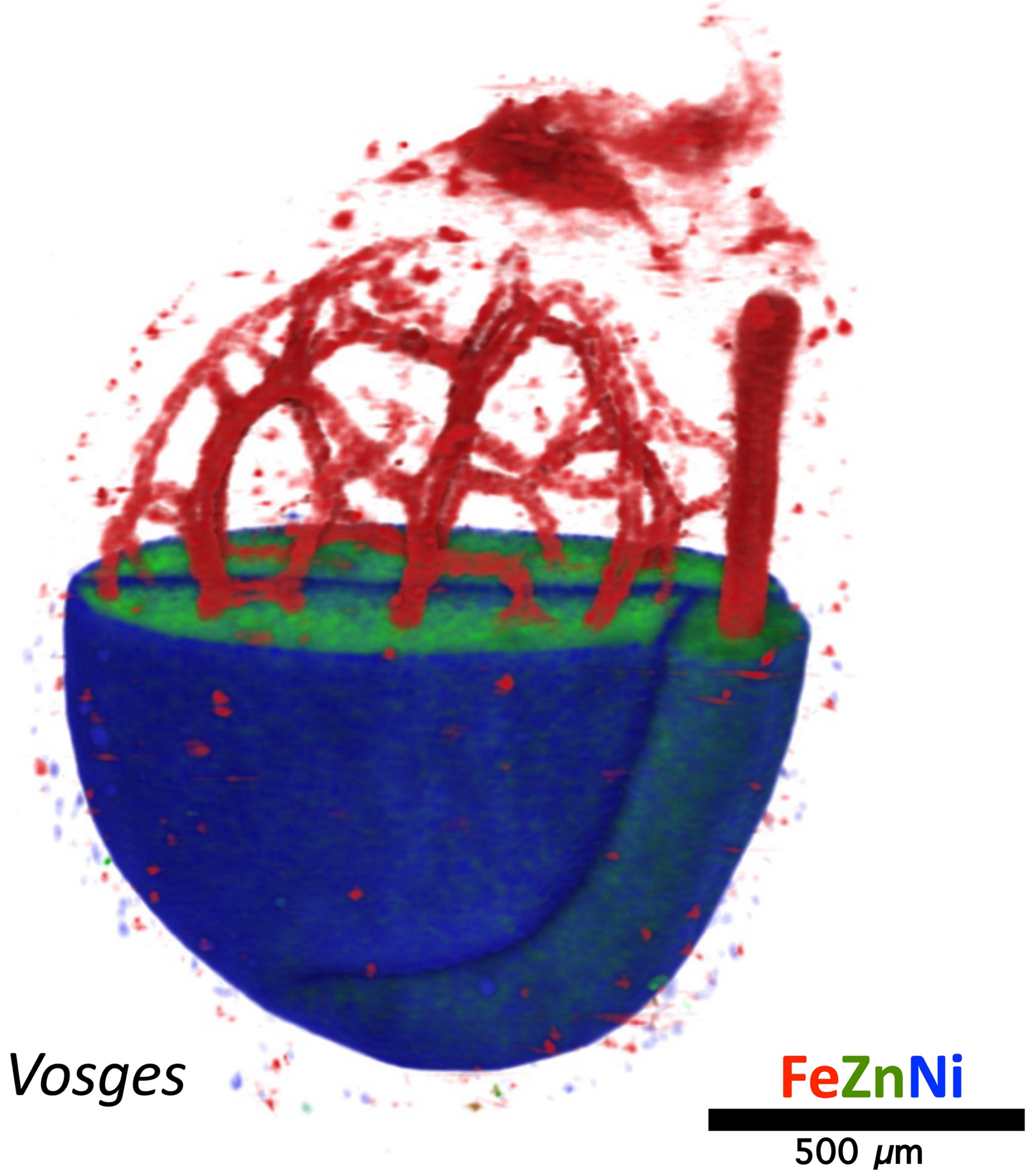

Suppl Fig 1. Tomographic reconstruction of dry *Noccaea caerulescens* seed showing Fe and Ni and Zn signals originating from the Vosges accession.

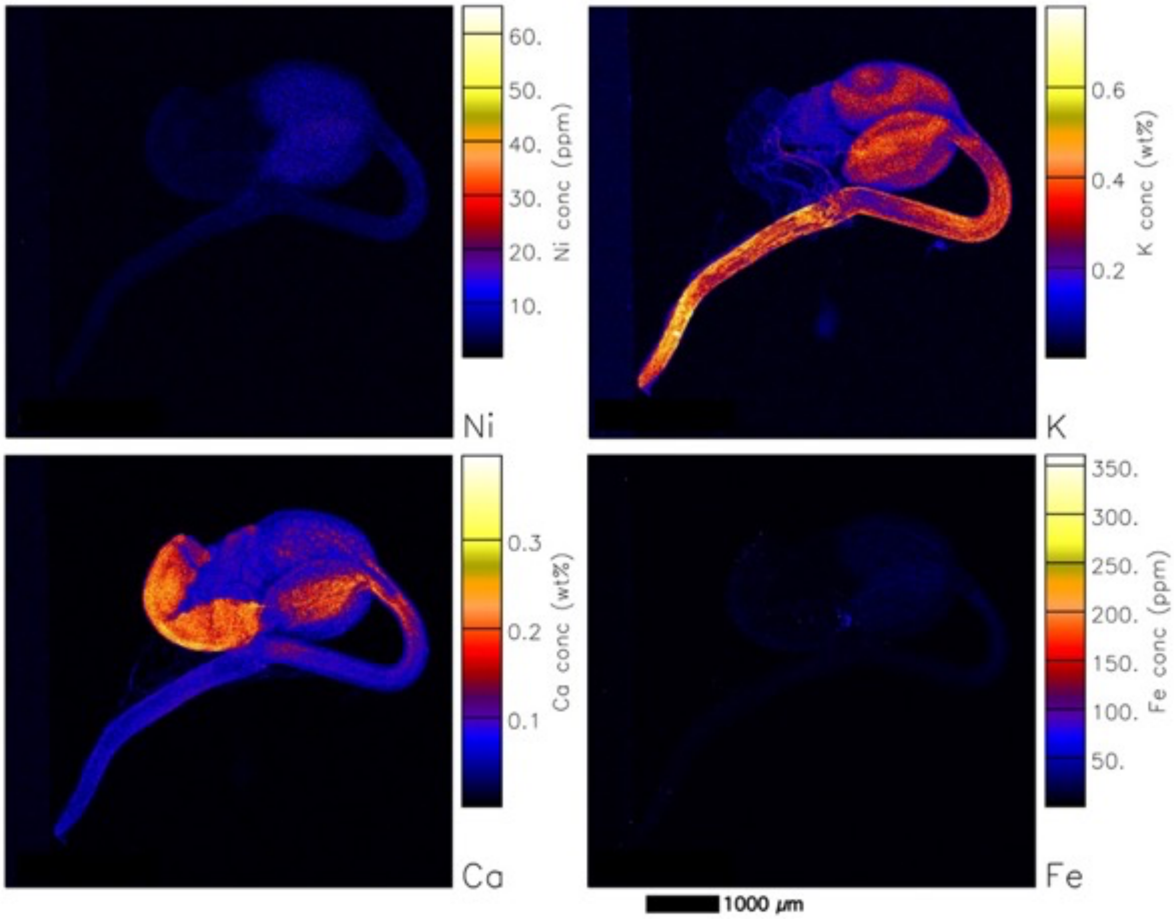

Suppl Fig 2. Elemental μXRF maps of living *Noccaea caerulescens* seedlings (Ganges, France accession). The scan size of the map is 21.2 × 30.8 mm (1245 × 1200 pixels). The elemental image was acquired in 5-μm step size with 2.5 ms dwell per pixel, 15.8 keV, incident beam, showing Ni, K, Ca and Fe maps.

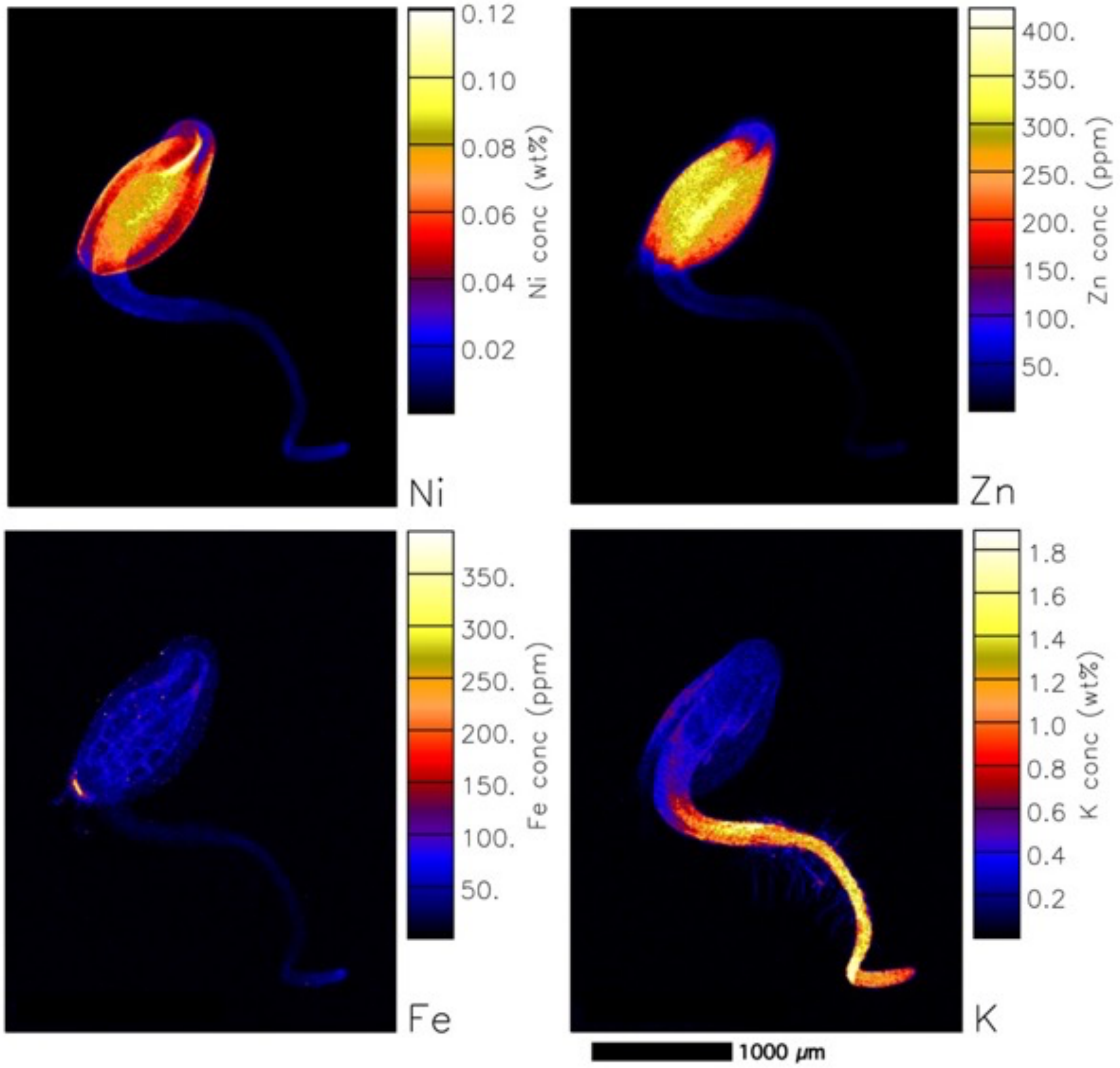

Suppl Fig 3. Elemental μXRF maps of living *Noccaea caerulescens* seedlings (Vosges, France accession). The scan size of the map is 22.6 × 8.8 mm (565 × 720 pixels). The elemental image was acquired in 5-μm step size with 2.5 ms dwell per pixel, 15.8 keV, incident beam, showing Ni, Zn, Fe and K maps.

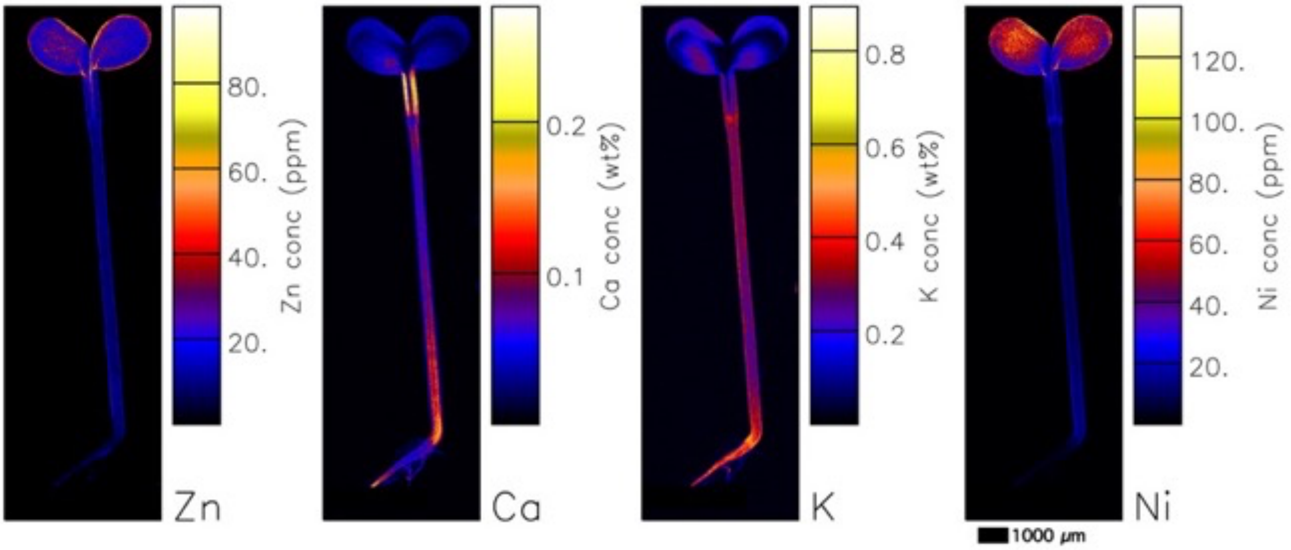

Suppl Fig 4. Elemental µXRF maps of living *Noccaea caerulescens* seedlings (Ganges, France accession**).** The scan size of the map is 39.1 × 40.6 mm (1050 × 3441 pixels). The elemental image was acquired in 5-µm step size with 1.7 ms dwell per pixel, 15.8 keV, incident beam, showing Zn, Ca, K and Ni maps.

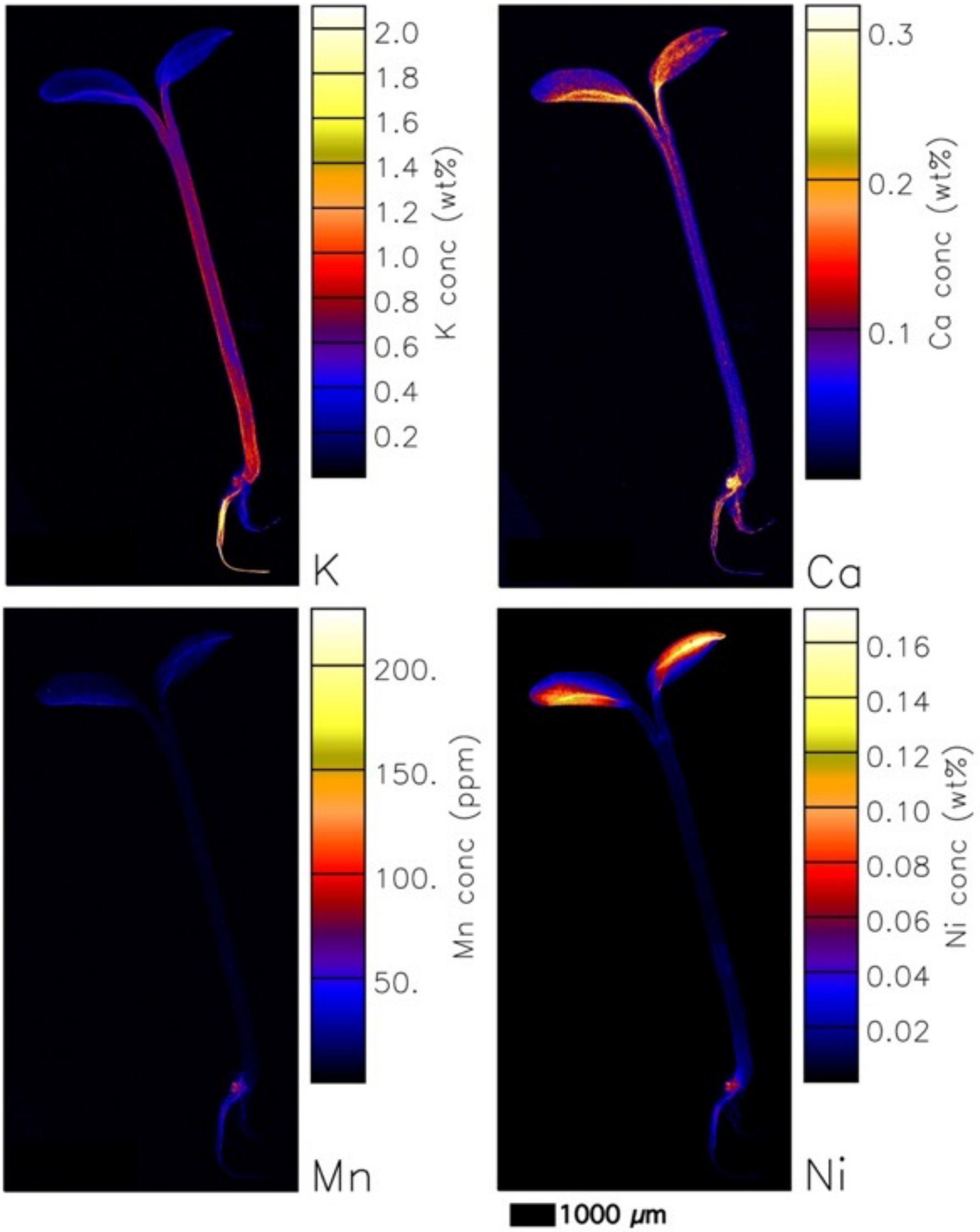

Suppl Fig 5. Elemental µXRF maps of living *Noccaea caerulescens* seedlings (Cira, Spain accession). The scan size of the map is 28 × 14 mm (1094 × 2168 pixels). The elemental image was acquired in 6-µm step size with 1.5 ms dwell per pixel, 15.8 keV, incident beam, showing K, Ca, Mn and Ni maps.

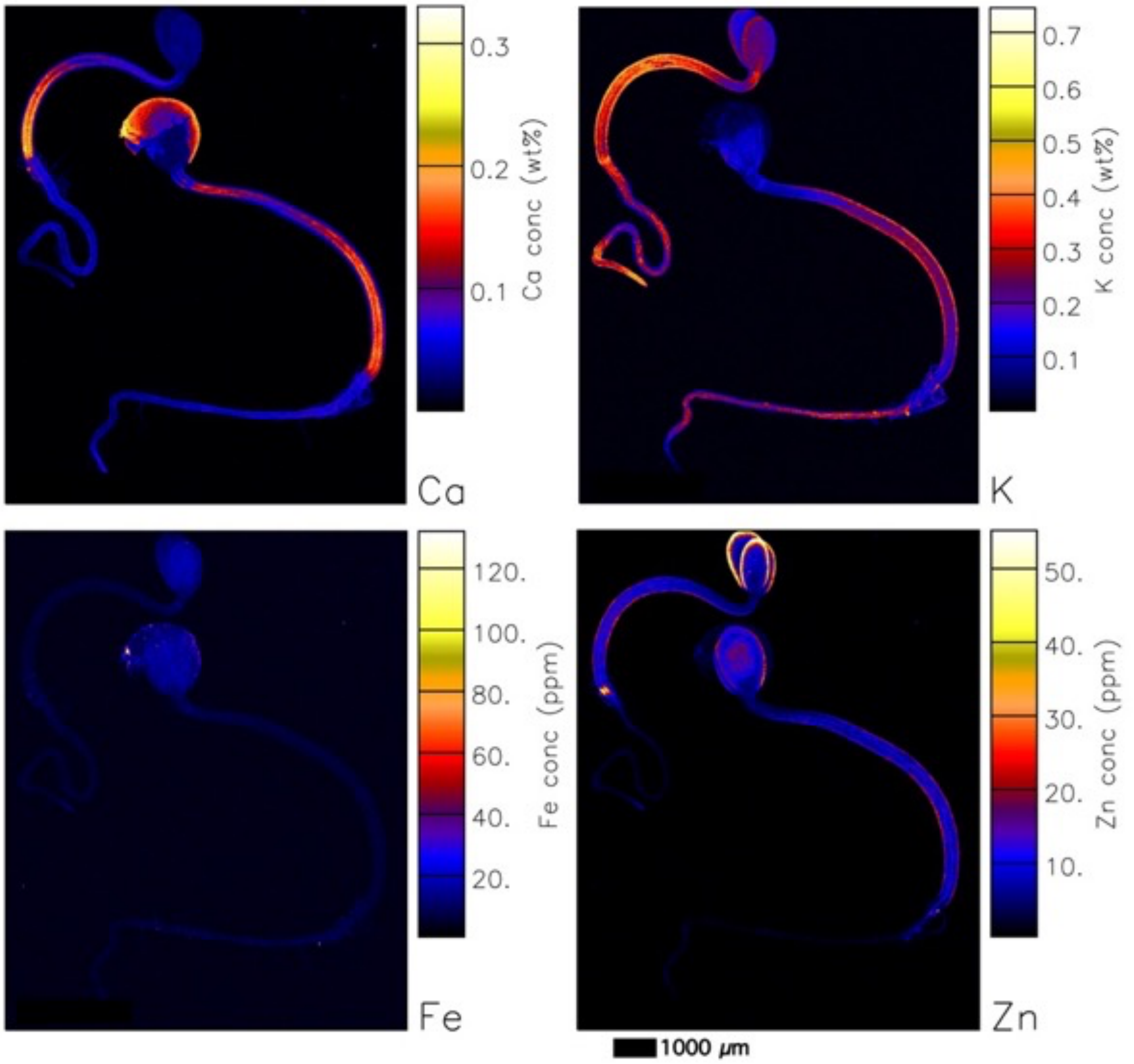

Suppl Fig 6. Elemental µXRF maps of living *Noccaea caerulescens* seedlings (Ganges, France accession). The scan size of the map is 43.9 × 44 mm (1928 × 2400 pixels). The elemental image was acquired in 5-µm step size with 1.7 ms dwell per pixel, 15.8 keV, incident beam, showing Ca, K, Fe and Zn maps.

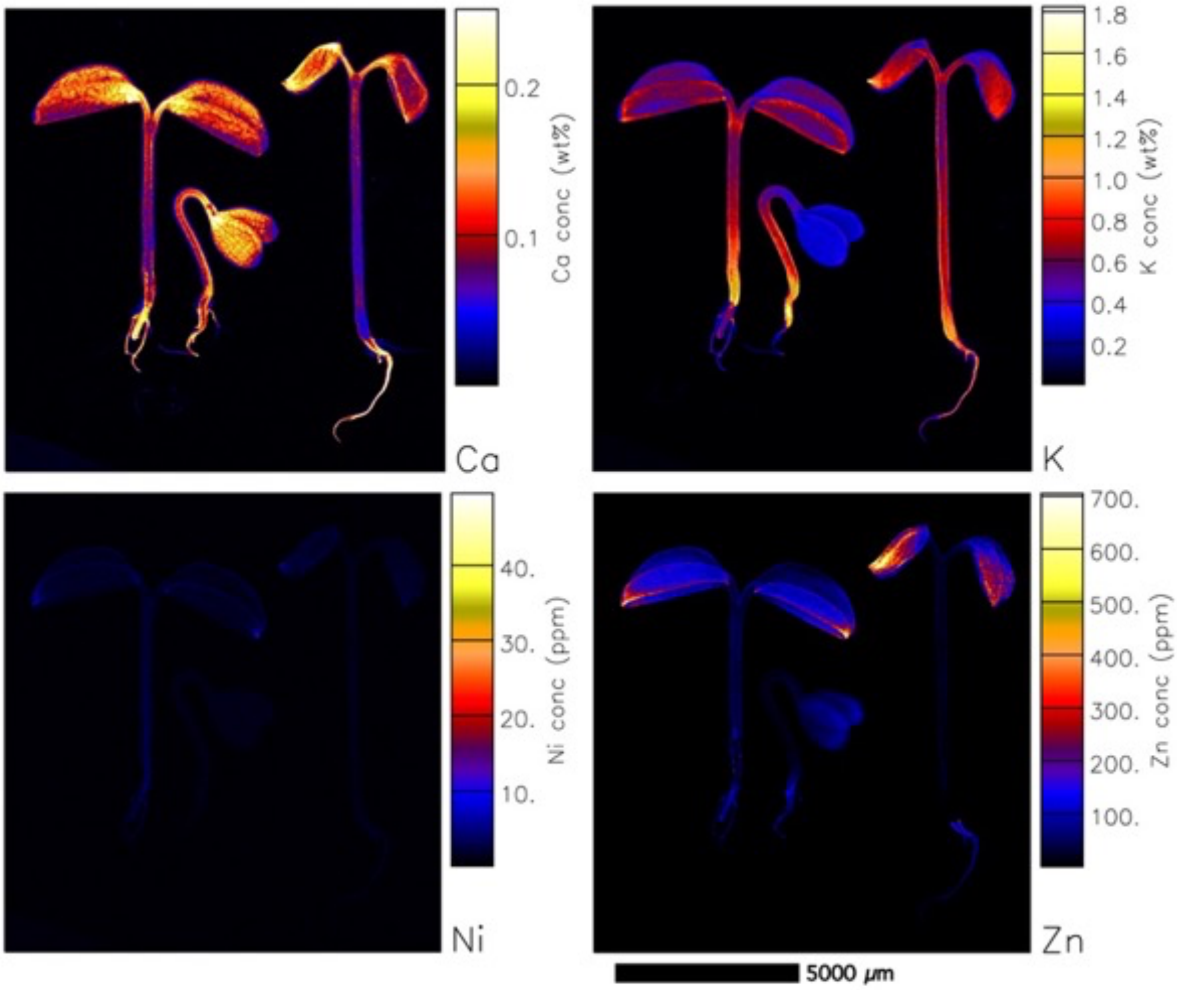

Suppl Fig 7. Elemental µXRF maps of living *Noccaea caerulescens* seedlings (Vosges, France accession). The scan size of the map is 20.3 × 40.2 mm (2368 × 2480 pixels), The elemental image was acquired in 5-µm step size with 1.7 ms dwell per pixel, 15.8 keV, incident beam, showing Ca, K, Ni and Zn maps.
